# Papillomavirus E5: the smallest oncoprotein with many functions

**DOI:** 10.1186/1476-4598-10-140

**Published:** 2011-11-11

**Authors:** Aldo Venuti, Francesca Paolini, Lubna Nasir, Annunziata Corteggio, Sante Roperto, Maria S Campo, Giuseppe Borzacchiello

**Affiliations:** 1 Laboratory of Virology, Regina Elena Cancer Institute, Rome, Italy; 2 Institute of Infection Immunity and Inflammation, University of Glasgow, Scotland, UK; 3 Department of Pathology and Animal health, University of Naples Federico II, Naples, Italy; 4 University of Glasgow, Glasgow, Scotland, UK

**Keywords:** Cell transformation, Growth factor receptors, Immune escape, Oncogene, Papillomaviruses, E5 oncoprotein, Animal models

## Abstract

Papillomaviruses (PVs) are established agents of human and animal cancers. They infect cutaneous and mucous epithelia. High Risk (HR) Human PVs (HPVs) are consistently associated with cancer of the uterine cervix, but are also involved in the etiopathogenesis of other cancer types. The early oncoproteins of PVs: E5, E6 and E7 are known to contribute to tumour progression. While the oncogenic activities of E6 and E7 are well characterised, the role of E5 is still rather nebulous. The widespread causal association of PVs with cancer makes their study worthwhile not only in humans but also in animal model systems. The Bovine PV (BPV) system has been the most useful animal model in understanding the oncogenic potential of PVs due to the pivotal role of its E5 oncoprotein in cell transformation. This review will highlight the differences between HPV-16 E5 (16E5) and E5 from other PVs, primarily from BPV. It will discuss the targeting of E5 as a possible therapeutic agent.

## Introduction

PVs are established agents of human and animal cancers [1]. They infect cutaneous and mucous epithelia inducing benign tumours which usually regress. Occasionally, the tumours progress to malignancy. Over 120 types of HPVs have been identified so far and among these 15 have been defined as HR HPVs. These are consistently associated with cancer. Genital HPVs are sexually transmitted and HR genital HPVs are a necessary factor in the development of almost all cases of cervical cancer. HPV-16 and -18 are the viruses most frequently associated with cancer of the uterine cervix (CxCa) [2].

CxCa is the second most common cancer in women worldwide killing about 0.25 million women per year. However, in economically developed countries the rate of CxCa is dramatically reduced due to screening program based on exfoliative cervical cytology (PAP smears).Vaccines to prevent HR HPV infection are available, although their use should be implemented along with screening programmes to further reduce the incidence of such cancer [3]. HR HPVs are also involved in the etiopathogenesis of other anogenital cancer [4]. Furthermore HPV, particularly HR HPV-16 is strongly associated to oral squamous cell carcinoma and other potentially malignant oral lesions [5]. Growing evidence also suggests that HR HPV-16 is involved in the etiopathogenesis of head and neck squamous cell carcinomas, suggesting that HPV vaccines should be also considered for prevention of this type of cancer [5,6]. Additionally, HPVs may be involved in the etiopathogenesis of others cancer types, including tumours of the upper respiratory tract, eye, esophagus, non-small-cell lung cancers [7-10]. The presence of HPV-16 has been reported also in colorectal carcinoma [11], breast cancer [12] and urinary bladder carcinoma [13]. Recently, HPV DNA has been associated also with prostatic tumours [14]. The widespread causal association of PVs with cancer makes their study worthwhile not only in humans but also in animal model systems which often provide new and profitable avenues of research [15]. The BPV system has been one of the most useful animal models in understanding the oncogenic potential of PVs. Furthermore, the mechanisms by which BPV induces tumors are an outstanding model to better understand the pathogenesis of other cancer types. The importance of the role of HPV in cancer etiology and development has been recognized by the assignment of the 2008 Nobel Prize for Medicine to Prof. Harald zur Hausen who firstly observed that infection with HPVs is responsible for CxCa development. The genome of PVs is a double stranded circular DNA roughly divided into three parts: the E region coding for early proteins (E1-E7) responsible for the pathogenicity of the virus; the L region coding for late structural proteins (L1, L2) and a non coding region which contains the cis-elements necessary for replication and transcription of the viral genome. Both in vivo and in vitro studies have pointed to E6 and E7 as the main HPV oncogenes, whereas E5 is the major oncogene of BPV. The E6 oncoprotein interacts with the cellular tumour suppressor p53 [16] and directs its degradation [17]. The primary target of the E7 oncoprotein is the retinoblastoma (Rb) proteins, the inactivation of which leads to tumour progression [18]. Both E6 and E7 also interact with many others cellular factors inducing genomic instability, tumour progression and immune evasion [18].

While the oncogenic activities of E6 and E7 are well characterised, the role of E5 is still rather nebulous. However, recent studies have highlighted the important role of this oncoprotein in cell transformation, tumourigenesis and immune modulation, thus implicating E5 in pivotal steps of carcinogenesis. Because of the perceived growing role of E5 in infection establishment and in cell transformation, it is worth considering the most salient features of the oncoprotein. This review will focus on the activities of E5, in particular from HPV-16, and from BPV. Targeting E5 as a possible therapeutic agent will also be briefly discussed.

## HPV E5

### E5 and the evolution of HPVs

HPVs have been classified into five genera (Alpha-, Beta-, Gamma-, Mu- and Nu-PVs), but not all HPV genera code for an E5 protein [19]. For instance αHPVs encode and express E5 but β HPVs do not. A phylogenetic analysis of known genital HPV types (derived using a Bayesian methodology, with notation of carcinogenic risk levels assigned by the large case-control study conducted by the International Agency for Research on Cancer [20]), suggested that at least 3 ancestral papillomaviruses are responsible for the current heterogeneous group of genital HPVs. The three major groups that emerged include alpha papillomavirus species and interestingly all carcinogenic types derive from a common ancestor and can code for an E5 protein, whereas the other HPVs either lack a definable E5 ORF (Open Reading Frame) or a translation start codon for E5. As notable exceptions, 10 types which cause benign venereal warts, can also code for an E5 protein. In addition, 16E5 variants with the greatest mitogenic activity *in vitro *[21] are most frequently detected in the population and most commonly associated with cervical lesions.

**Figure 1:**
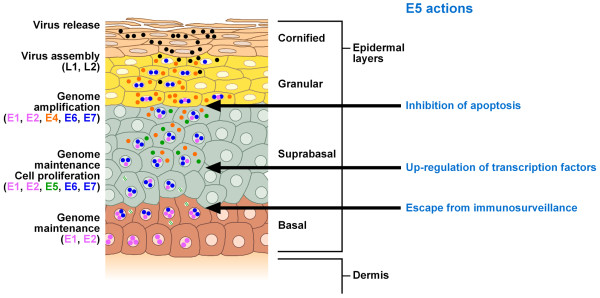
**E5 can improve HPV activity by altering host factors controlling the viral replication/persistence**. A schematic view of the papillomavirus life cycle highlighting the expression of the HPV genes, which is tightly regulated and strictly linked to epithelial differentiation. E5 could contribute to a successful infection by inducing loss of surface MHC I expression in the infected basal cells preventing presentation of viral antigens to effector T-cells and thus, in addition to other mechanisms of immune avoidance, such as lack of inflammation, contributing to evasion of immune surveillance. Expression of E5 in the basal/suprabasal layers of the epithelium would lead to sustained cell proliferation to favour virus-infected cells, but extinction of its expression in the more superficial layers would permit cell differentiation and virion production. If E5 expression proceeds beyond early lesional stages, keratinocyte differentiation and immunological removal of infected cells would not take place and the lesion would be at greater risk for neoplastic progression. E5 actions on host factors controlling viral replication/persistence are indicated. Dashed dots indicate low levels of E5 gene expression.

The HPV E5 ORF itself has been classified into four different groups: alpha, beta, gamma and delta [22], which correlate with different clinical manifestations, in particular with oncogenic potential [23]. Thus, the E5-alpha protein is encoded by HR αHPV, whereas the E5-gamma and E5-delta proteins are encoded by low-risk genital HPVs [24]. The phylogenetic analysis suggests that E5 must give some advantage to the virus expressing it and variants of this protein appear to increase the likelihood of oncogenic transformation following persistent infections. However, the E5 ORF is absent in the genome of many HPVs, such as beta-, gamma- and mu-HPVs, indicating that the protein is not essential for the life cycle of these viruses but rather can give some ADDED VALUE to favour infection and transformation (Figure 1). The HPV E5 ORF is expressed during the early phases of the viral life cycle but only as the fourth ORFs on polycistronic transcripts. Since HPVs are thought to use a leaky ribosome-scanning mechanism to translate proteins from polycistronic mRNAs, little E5 protein is likely to be synthesized from these transcripts. In contrast, on epithelial-cell differentiation, E5 is expressed as the second ORFs of late transcripts. It is therefore likely that E5 is synthesized highly in differentiating suprabasal epithelial cells [25] (Figure 1). In agreement, the BPV-1 E5 protein was detected at a low uniform level in basal layers and at a higher level in the uppermost layers of stratified squamous epithelium in papillomas productively replicating BPV-1 [26]. In addition, E5 was also shown to be expressed in basal and suprabasal layers of BPV4-induced papillomas [27] and of HPV-16 induced cervix lesions [28].

In contrast to the highly transforming BPV-1 E5, the HPV E5 proteins display weak transforming activity *in vitro *(Table 1). Experiments with HPV-6 provided the first evidence that a HPV E5 protein had transforming activity in mammalian cells, as expression of HPV-6 E5 in established murine fibroblasts lead to anchorage independent growth [29]. Later it was shown that also 16E5 induces anchorage independence, more efficient growth in low serum and tumorigenic transformation of murine keratinocytes and fibroblasts [30-32]. In addition, the acute expression of 16E5 stimulates cellular DNA synthesis in primary human keratinocytes, and in cooperation with E7, induces proliferation of primary rodent cells [33-36]. The transforming activity of E5 from HPV-59 and rhesus papillomavirus has been demonstrated in various cell types and assays [37,38].

**Table 1:** Comparison of function of HPV E5 proteins with BPV E5 proteins.

E5	LR HPV-6	HR HPV-16	BPV-1	BPV-4
FF	n.e.	-	+	+

SI	n.e.	+	+	+

AI	+	+	+	+

Koilocytosis	+	+	n.e.	n.e.

PDGFβR activation	n.e.	-	+	n.e.

EGF-R activation	+	+	+	n.e.

V-ATPase binding	+	+	+	+

Gap-j inhibition	n.e.	+	+	+

PI3-K activation	n.e.	+	+	n.e.

c-Src activation	-	-	+	+

Cyclin-cdk2 activation	n.e.	+	+	++

MHC I down-regulation	+	+	+	+

KNβ3 binding	n.e.	+	n.e.	n.e.

TRAIL pathway inhibition	n.e.	+	n.e.	n.e.

MAPK activation	+	+	-	n.e.

ETA activation	n.e.	+	n.e.	n.e.

PGE2 R expression	n.e.	+	n.e.	n.e.

Cellular zinc imbalance	n.e.	+	n.e.	n.e.

ER Stress pathway inhibition	-	+	n.e.	n.e.

Cellular fusion	-	+	n.e.	n.e.

P21 inibition	+	+	n.e.	n.e.

FF: focus formation; SI: serum independence; AI: anchorage independence. +: presence of function; -: absence of function; n.e.: not established.

### Structure and cell interaction

16E5 is 83 amino acid long. The detection of this protein has proved very difficult given its extreme hydrophobicity, membrane localisation and very low levels of expression. For these reasons expression of E5 is often inferred from the presence of E5 mRNA. 16E5 has been detected by immunohistochemistry by Chang et al. [28] in low-grade squamous intraepithelial lesions (LSILs), in high-grade SILs, CIN (cervical intraepithelial neoplasia) and paradoxically in cancer lesions although often this gene is deleted during viral integration into the host genome. Amino acid sequence analyses of 16E5 suggest it comprises 3 anchor-like α-helices (residues 8-30, 37-52 and 58-76), with only the first being long enough to span a lipid bilayer.

When over-expressed, 16E5 is present in the endoplasmic reticulum (ER), in the nuclear envelope and in the Golgi apparatus (GA) [39], whereas at a more physiological level of expression, as shown in primary human foreskin keratinocytes, it localizes almost exclusively to the ER and to lesser extent to GA and early endosomes [40]. Recently, it was reported that in HaCaT cells about 10% of total 16E5 can localize to plasma membrane with intracellular amino terminus and extracellular carboxyl-terminus. This observation suggests a fusogenic role of 16E5 with the induction of cell-cell fusion and the formation of binucleated HaCaT cells [41-43]. In contrast, other authors report the presence of 16E5 protein only in the ER of COS and ectocervical cells in the opposite orientation: intraluminal amino-terminus and cytoplasmic carboxyl-terminus [44]. The cytoplasmic localization of the C terminus is further strengthened by the reported interaction of 10 amino acids at the C terminus of 16E5 with karyopherin β3 (KNβ3) [45], an abundant cellular protein localized mainly in the cytoplasm [46,47]. An explanation for these contrasting data is the possible presence of 16E5 in the plasma membrane as a consequence of the over-expression of 16E5 by the adenovirus vector system used in HaCaT cells. In agreement, when BPV-1 E5 is expressed at very high levels, such as in baculovirus-infected cells, it is detectable on the plasma membrane in addition to the ER and GA [48].

16E5 self-associates *in vitro *[49] and *in vivo *[40,50] and this oligomerization takes part mostly by hydrophobic interaction. The reported disulfide bonding [40] appears less likely because models of 16E5 membrane topology predict the localization of all six cysteine residues within the lipid bilayer [21,51] rendering cysteine dimerization more difficult.

### E5 and cell transformation

E5 interacts with a number of cellular proteins and these interactions are deemed important for the biological activity of the protein in cell transformation and evasion of the immune response. Thus the first transmembrane domain of 16E5 and HPV-31E5 interacts directly with the heavy chain component of the MHC I (Major Histocompatibility Complex class I) via the leucine pairs present in this region; interestingly this same transmembrane domain interacts with a chaperone of MHC I, Bap31 [52,53]. A region within the second helix (residues 41-54) may be the binding site for the 16 kDa pore sub-unit of vacuolar-ATPase (V-ATPase) [54], although others claim this is located between residues 54-78 [55].

E5 is a weak transforming protein *in vitro *and its effects are seen best when in co-operation with the other viral oncoproteins: 16E5 together with E6 can induce the formation of koilocytes, large cells with cleared cytoplasm and pyknotic nuclei with inconspicuous nucleoli, a well known morphological marker of HPV infection [56]. The development of koilocytotic vacuoles may be linked to the E5-induced relocalization of calpactin I to the perinuclear region promoting perinuclear membrane fusion [57]. Hu et al. [43] confirmed previous reports describing the oncogenic capacity of 16E5 [30,58] and offered mechanistic insights into how E5 expression brings about morphological changes in the cervical epithelium. Further, by identifying endoreplication as the mechanism by which the aberrant nuclei form and increased DNA synthesis arises, there is now a biological process that can be targeted to inhibit oncogenesis [43]. Fusogenic 16E5 is expressed on the plasma membrane of cells and both cells must express E5 for cell-cell fusion to occur, indicating that E5 cannot induce cell-cell fusion through an interaction with another protein (receptor) on a neighbouring cell, but rather forms E5-E5 dimers or complexes containing at least two 16E5 molecules. These findings provide important insight into how the fusogenic process is mediated [41].

E5 of HR HPV by itself can induce morphological and chromosomal changes that frequently accompany the progression of normal cells to cancerous cells. Increased nuclear size, increased DNA content and tetraploidy are characteristic of LSIL [59,60]. All of these morphological changes have been detected in cell expressing 16E5 and, in particular, the aberrant nuclei formation seems to be due to endoreplication rather than a consequence of cell-cell fusion and failed cytokinesis [43]. Many of these changes are criteria used in the clinical detection of cervical cancer precursors in screening Pap tests [61]. Other morphological changes, such as the presence of binucleated cells, increased DNA content and polyploidy [62], are not used in the diagnosis of precancerous lesions despite being less subjective because they require larger amounts of material, time, and expenses.

### HPV E5 interaction with host cell

HPVs infect stratified epithelia, and their whole life cycle is inextricably linked to keratinocyte differentiation and, in order to establish a persistent infection, HPVs have evolved to overcome many "obstacles":

a) The stratified epithelium is associated with continuous cellular turnover and desquamation of the terminally differentiated keratinocytes, thus maintenance of the papillomavirus within the tissue requires the infection of basal epithelial cells and the propagation of these infected cells.

b) Papillomaviruses use the cellular machinery for their replication and need to maintain cell division together with a delayed but not completely inhibited differentiation. HPV infected keratinocytes with imbalanced DNA synthesis/differentiation are forced to apoptosis by the cellular control mechanism.

c) To establish persistent infection HPV must fight or evade immune-surveillance.

It is clear that the timely and epithelial differentiation-dependent expression of all E proteins is essential to favour viral replication and in turn to overcome the above mentioned obstacles. The "E5 added value" will be highlighted in the following paragraphs.

### E5, growth factors and cell cycling

**Figure 2:**
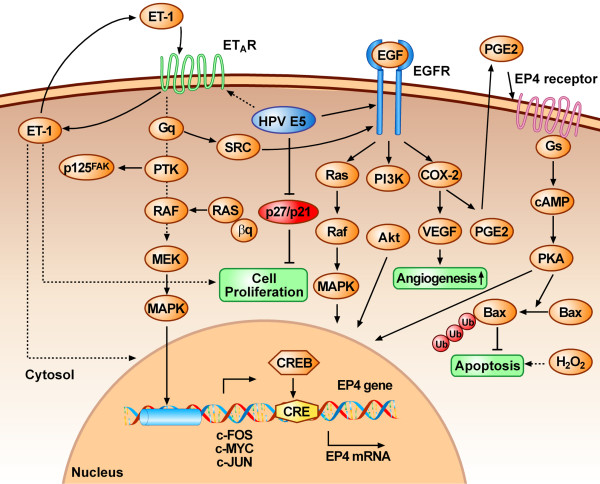
**HPV 16 E5 enhances growth factor signalling pathways**. Activation of EGF-R and the downstream Ras-Raf-MAP kinase pathway or PI3K-Akt pathway leads to altered cell proliferation, angiogenesis, and anti-apoptosis. The last two functions are further enhanced by the E5-induced upregulation of COX-2 expression. COX-2 expression inhibits hydrogen peroxide-induced apoptosis and induces PGE2 secretion that activates EP4 receptor. This, in turn, causes cAMP production, activating PKA; PKA contributes to E5-induced expression of EP4 by enhancing CREB binding to variant CRE of the EP4 promoter. PKA influences also apoptosis through the ubiquitin-proteasome degradation of BAX. Finally, E5 protein enhances cell proliferation through downregulation of tumor suppressor p21/p27 and through up-regulation of G protein-coupled endothelin receptor (ET_A_)/ET1 autocrine loop. Dotted arrow shafts indicate uncertain pathways. The possible cross-talking between ETAR and EGFR pathway through Src protein is also indicated.

HPV infection is thought to take place when epithelial basal cells are directly exposed to the virus during microinjuries [63]. Infection of epithelial stem cells, which have the capacity for self-renewal, can ensure long term maintenance of the viral genome. During the productive stage of the HPV life cycle, the early viral proteins are expressed, maintaining 50-100 copies of episomal DNA per cell by synchronous replication with host cell DNA [64-66]. The demonstrated ability of 16E5 to enhance ligand-dependent activation of the EGF-R [56,58-60] (Figure 2) and to stimulate EGF-dependent proliferation of cultured human keratinocytes [33,35,58] suggests that 16 E5 may play a major role in expanding populations of HPV-16-infected basal keratinocytes *in vivo *by enhancing ligand-dependent EGF-R activation. Confirmation of this E5 activity comes from a study on 16E5 transgenic mice showing that functional EGF-R is required for the induction of epidermal hyperplasia and formation of spontaneous skin tumours [67]. 16E5 oncoprotein binds and inhibits the activity of the 16 kDa subunit of V-ATPase, altering the endosomal acidification and degradation of EGF-R [34,68] thus enhancing its recycling to the plasma membrane. 16E5 can also delay EGF-R degradation by interfering with membrane trafficking and the fusion of early and late endosomes [69]. Recently, 16E5 was shown to form a complex with KNβ3 which is localized mainly in the cytoplasm near the nuclear envelope [45]. It has been suggested that a KNβ3/16E5 complex plays an important role in vesicle trafficking, reinforcing the hypothesis that E5 has a central role in altering protein trafficking inside the cell. The EGF-R signalling pathway can be activated by 16E5 through either EGF-dependent or EGF-independent processes. 16E5 activates mitogen-activated protein kinase (MAPK) p38 and ERK1/2 in human keratinocytes in an EGF-independent manner [70]. Two different pathways, a receptor tyrosin kinase-mediated pathway and a protein kinase C (PKC)-dependent pathway, are involved in the MAPK activation [71,72] which increases the transcription of *c-fos *and *c-jun *[33,73,74], forcing the cells through the cell cycle and stimulating transcription of the viral oncogenes E6 and E7. In contrast with EGF-R data, less is know about the role of the interaction of 16E5 with other ErbB family members such as ErbB2 or ErbB4 receptor [28,75-77].

16E5 is also capable of interacting with, and enhancing the signalling of, different classes of growth factor receptors like the G protein-coupled endothelin receptor (ET_A_) [36]. In growth factor-starved keratinocytes 16E5 enhances the mitogenic activity of endothelin-1 (ET-1) the specific ligand of ET_A _(Figure 2). Interestingly it was reported that the mitogenic activity of ET-1 may lead to the chronic stimulation of keratinocyte proliferation observed in psoriasis, an inflammatory/proliferative disorder of the skin, suggesting an important role for ET-1 in epithelial proliferation [78]. Furthermore cross-talking between ET_A_R and EGF-R pathways would have an amplifying effect on cell proliferation [79].

In contrast to the activation of EGF-R, 16E5 can down-modulate the keratinocyte growth factor receptor/fibroblast growth factor receptor 2b (KGF-R/FGF-R2b), through reduction of transcripts and protein with alteration of the receptor endocytic trafficking. This causes a decrease in the growth response to the receptor ligands, suggesting that 16E5 might have a role on HPV infection by perturbing the KGFR-mediated physiological behaviour of confluent keratinocytes committed to differentiation [80]. Thus, 16E5 could exert two opposite effects, both related to virus replication: (a) increasing the growth of the most basal undifferentiated cells by up-regulating the EGF-R pathway and (b) decreasing the physiological proliferation/differentiation of the suprabasal keratinocytes by down-modulation of KGFR expression and signalling.

16E5 can also down-regulate the expression of tumour suppressor p21 and p27, both of which are cyclin-dependent protein kinase inhibitors (CKIs), thus causing cell cycle progression and DNA synthesis (S-phase) (Figure 2). The p21^Wafl/Sdil/Cipl ^down-regulation is at transcriptional level [81] whereas that of p27^Kip1^, one of the most abundant CKIs, is through reduction of the half-life of p27^Kip1 ^protein [82].

Other biological activities of 16E5 contribute to the proliferation of the infected cells, such as the interference with cell-cell communication and alteration of adherence and cell motility. The ability of 16E5 to inhibit gap junction-mediated communication between epithelial cells in monolayer [83] and in raft cultures [84] by interfering with connexin 43 may render the transformed cells more insensitive to homeostatic growth control signals from adjacent normal cells.

### E5 and apoptosis

Due to the integration of HR HPV genome during malignant progression, the E5 gene is not expressed in cervical tumours but both 16/18 E5 mRNA and protein have been detected in anogenital LSIL [85,86] supporting the possibility that E5 plays a role in early steps of HPV infection to protect infected cells from apoptosis. Indeed, it has been proposed that inhibition of death receptor-mediated apoptosis in human keratinocytes, needed to prevent apoptosis at early stages of viral infection, is a primary function of the HR HPV E5 protein. 16E5 impairs tumor necrosis factor ligand (FasL) and Tumor necrosis factor-related apoptosis-inducing ligand (TRAIL)-mediated apoptosis in HaCaT cells by: (a) downregulating the total amount of Fas receptor and reducing Fas surface location; and (b) altering the formation of Death-Inducing Signalling Complex (DISC) triggered by TRAIL [87]. Raft cultures of 16E5-expressing keratinocytes were completely protected from FasL- or TRAIL-induced cell death [88]. Likewise, when UV radiation was used to induce stress, E5-expressing human keratinocytes were protected from apoptosis [89]. In contrast, 16E5 sensitizes human keratinocytes to apoptosis induced by osmotic stress, perhaps due to cell membrane modifications caused by this strongly hydrophobic molecule [90].

### E5 and ER stress pathway

The presence of viral proteins may activate cellular defence mechanisms and in particular the ER stress response. 16E5 can suppress three key proteins of the ER stress pathway: cyclooxygenase-2 (COX-2), XBP-1 and IRE1a. As it was suggested for other viruses, the down-regulation of these proteins can favour viral persistence [91] which is a major contributory factor to the development of cancer by high-risk HPVs [92]. The inhibition of the ER stress pathway by E5 seems to be limited to the high risk types (Table 1); HPV-6b E5 is unable to alter XBP-1 [93] and increased levels of COX-2 were reported in recurrent respiratory papillomatosis (RRP) lesions caused by low-risk HPV type 6b and 11 [94,95]. 16E5 is also able to lower COX-2 expression in cells co-expressing E6/E7, suggesting that it might exert similar activity during viral replication.

However, in different cell systems and clinical conditions the same viral early genes seem to exert opposite effects such as the induction of COX-2 expression in spontaneously immortalized HaCaT and cervical cancer lines C33A and SiHa [96,97]. It is possible that the different cell origin, immortalization and transformation status, and the relative expression of the HPV early proteins account for these contrasting data. Nevertheless the reported consistent down-regulation of ER stress response genes by 16E5 in primary genital keratinocytes leads to speculation that this ER stress pathway inhibition is an event favourable to viral replication and persistence [98].

Finally, 16E5 may induce expression of one of the Prostaglandin E2 (PGE_2_) receptor in cervical cancer cells by stimulating the binding of CREB to a variant CRE site in the promoter of EP4 gene [99] (Figure 2). EP4 pathway activates protein-kinase A which mediates ubiquitin-proteasome-mediated Bax degradation, inducing antiapoptotic effects. Activation of EP4 by 16E5 increases anchorage-independent colony formation and vascular endothelial growth factor (VEGF) expression, leading to speculation that al least in some tumours E5 is involved in tumour growth, angiogenesis and metastasis by inducing inflammatory cell signalling pathways.

### HPV E5 and immune evasion

Cervical carcinogenesis is a multi-step process which starts with viral infection; one of the steps is persistence of viral infection. At least three major factors may favour papillomavirus persistence: the virus life cycle takes place away from dermal immune cells [100], the virus does not cause cell lysis and therefore no or weak inflammatory response [100], and, finally, the viral proteins actively fight the immune response [101]. The E6 and E7 proteins play an important role in this fight but, once again, the E5 protein seems to add help to the virus by down-regulating MHC/HLA class I. MHC class I is much reduced on the cell surface and accumulates in the GA in cells expressing 16E5 [102]. The arrest of MHC class I in the GA is due to E5-induced alkalinisation of the endomembrane compartments [103], following 16E5-16 k subunit c interaction, and to the direct interaction of E5 with the heavy chain of the MHC class I complex [53,104]. These conclusions have been reached in cultured HaCaT cells and W12 cell line, and *in vivo *in the bovine model [105]. The functional effect of the decreased expression of HLA is a reduction of the recognition by CD8^+ ^T cells in vitro [106].

Furthermore consistent with its role in the alkalinization of endosomes, 16E5 can prevent the endosomal breakdown of the invariant chain, a chaperone important in the maturation of HLA class II, leading to inhibition of expression of surface HLA class II [107].

Inhibition of HLA class I transport by 16E5, in contrast to inhibition by BPV E5, is reversible by interferon (IFN) treatment. IFN is sufficient to overcome the inhibitory effect on MHC transport in presence of low levels of 16E5. However, in oncogenic HPV infections, E6 and E7 can inhibit the type I IFN pathway [108-110], thus preventing the IFN-mediated release of E5-induced blockage of HLA class I traffic.

Although an efficient mechanism to avoid cytoxic T lymphocytes (CTL)-mediated immune clearance, the reduction or absence of surface MHC class I renders cells more susceptible to Natural Killer (NK) cell attack. Human NK cells express multiple receptors that interact with HLA class I molecules, including killer cell immunoglobulin-like receptors (KIRs) that predominantly recognize classical HLA class I, including HLA-C, and the C type lectin superfamily of receptors that specifically interact with the nonclassical class I molecule HLA-E. Recognition of the class I molecules by their inhibitory receptors inhibits NK-mediated cell lysis, which would occur in the absence of HLA-C/E. Thus to efficiently evade immunosurveillance, HPV has to selectively down-regulates the HLA class I molecules. Indeed 16E5 selectively inhibits surface expression of HLA-A and HLA-B without affecting either synthesis or transport to the cell surface of HLA-C/E [97,100,101]. In this way HPV-16 is potentially capable of avoiding both CTL and NK cell killing.

The bridging of innate and adaptive immune responses can also be affected by E5 via the inhibition of CD1d-mediated cytokine production that would otherwise occurs upon interaction between cell surface CD1d and iNKT cells [111].

It was reported that interactions between 16E5 and calnexin interfere with modification of HLA class I HCs and results in heavy-chain retention in the ER [51]. Since the synthetic pathways for CD1d and HLA class I HCs are similar, this E5-calnexin interaction may alter CD1d trafficking. Indeed, interactions between 16E5 and calnexin do not appear to interrupt all of the functions of calnexin, but just enough to coopt the cellular cytosolic proteolytic pathway and degrade CD1d. Thus E5 inhibits the CD1d-mediated innate and adaptive immune pathways early in HPV infection.

Finally, the reported novel association of HPV-16 and HPV-31 E5 with Bap31 and A4 can also have an effect on immune escape [52]. Bap31 is a chaperone involved in quality control of, for instance, MHC molecules; A4 is a putative ion channel protein of the endoplasmic reticulum. E5 and Bap31 physically interact and colocalise in perinuclear structures and it was demonstrated that this binding correlates with the ability to retain the proliferative capacity of infected keratinocytes following differentiation. E5 also binds and colocalises with A4 independently of Bap31, however the biological significance of this interaction remains to be established. Interestingly 16E5 first transmembrane domain which binds MHC heavy chain shows homology with the third transmembrane domain of Bap31 [53]. Therefore it is possible that membrane-bound 16E5 displaces Bap31 from MHC I, maybe taking advantage of its own interaction with Bap31, and thus retains MHC I in the ER/GA.

Thus immune escape mediated by E5 seems to be a complex process, perhaps evolved to impact on different pathways regulating the intracellular trafficking of immunosurveillance molecules by general not-specific mechanisms, such as endosomal alkalization, and specific ones, such as binding to MHC I heavy chain and chaperones such as calnexin, invariant chain or Bap31. It remains to be seen if E5 expression causes immune escape also *in vivo*. An inverse correlation exists between expression of 16E5 and presence of surface MHC I in the W12 keratinocyte line derived from a CIN I biopsy [106], and in a small panel of naturally occurring CIN lesions (Campo, unpublished observations). Additionally, again in a limited number of samples, an association has been reported between decreased CD1d immunoreactivity and progression of cervical neoplastic lesions with statistical significance (*P *= 0.0001) [111].

### Cell transformation pathways alternative to E5

Beta-HPVs do not code for an E5 protein. Considering the ubiquitous presence of beta-HPVs in the healthy population with almost no associated pathologies, the lack of E5 ORF in these HPV could represent a factor hampering the fully replicative cycle of these viruses. Moreover, some beta-HPVs are detected together with HPV-3 or related genotypes [112], and such a co-detection with E5-encoding HPVs suggests that beta-HPVs benefit from E5 delivered by the co-infecting HPV [113]. In human pathology there is a rare, autosomal recessive genodermatosis associated with a high risk of skin carcinoma, Epidermodysplasia Verruciformis (EV), characterized by abnormal susceptibility to infection by beta HPVs. Genetic analysis led to the identification of two adjacent genes (EVER1 and EVER2), the mutation of which segregates with the disease [114]. This extraordinarily high sensitivity to infections by cutaneous beta-HPVs in otherwise-healthy individuals carrying a mutation in one of the EVER genes suggests that in humans a natural EVER-based barrier exists, which protects the host from PVs. The EVER proteins are crucial for the functional integrity of the EVER/ZnT-1 complex [115,116] responsible for maintaining a low level of free zinc, modulating the activity of the AP-1 transcription factors needed for viral genome expression. It has been proposed that a cellular zinc imbalance constitutes an important, perhaps even a crucial, step in the HPV life cycle. It seems that breaking the barrier to cellular zinc balance is an important element of the pathogenesis of both alpha- and beta-HPVs, and the main difference between these two groups would be a mechanism employed to achieve the same goal. Beta-HPVs are defective for this zinc imbalance, an important growth-promoting function performed by E5 of alpha-HPVs, and inactivation of EVER proteins may compensate for the missing viral function [117]. Indeed the transmembrane viral 16E5 and cellular EVER proteins interact both with the zinc transporter ZnT1, and are likely to modulate zinc homeostasis [115,117]. The disruption of the cellular zinc-transporting complex achieved during the long co-evolution of HPV and the human species by two completely unrelated strategies underlines the general importance of the cellular zinc (im)balance in the papillomavirus life cycle and the central role played by E5.

In the absence of E5, beta-papillomaviruses have evolved a mechanism to escape immunosurveillance independent of E5. Indeed the E6 and E7 oncoproteins of cutaneous HPV-38 interfere with the interferon pathway. Expression of the two viral proteins in HaCaT keratinocytes led to a decrease of MHC I levels. This down-regulation was associated with a reduction of expression of MHC I heavy chain, of the peptide chaperone TAP and of the STAT-1 downstream effector IRF-1 [118]. Thus, at least for some (high risk?) beta papillomavirus other E5-unrelated mechanisms take part in the process of viral replication and avoidance of immune control.

### HPV E5 and carcinogenesis

HPV E5 proteins are not thought to play a role in the later steps of malignant progression because in high-risk HPV infections that progress to cancer the viral DNA typically integrates into the host genome often resulting in the loss of the E2 and E5 genes [65]. However in contrast to other high-risk HPVs, HPV-16 DNA can exist in integrated, episomal or integrated and episomal form in malignant lesions of the cervix. In one study about 60% of HPV-16-positive cervical cancer expressed the 16E5 protein [28] and recent reports suggest that these tumours with episomal HPV-16 may have a more aggressive behaviour (Venuti, unpublished data). Nevertheless, the fact that a substantial proportion of the tumours do not express E5 indicates that the protein is not essential for HPV-16 mediated tumour progression.

Although 16E5 may not contribute to malignant progression, there is evidence that if E5 expression proceeds beyond early lesional stages, keratinocyte differentiation and immunological removal of infected cells does not take place increasing the likelihood of subsequent oncogenic transformation.

The presence of an E5 gene in all HR HPVs [23] and the detection of E5 variants with higher codon usage in high grade lesions [119] point to an important role of E5 during PV-induced pathogenesis. This assumption is further strengthen by studies on transgenic animals: (a) Transgenic mice expressing only HPV-16 E6 and E7 oncogenes in the basal epithelium develop fewer tumours than those arising in transgenic mice expressing HPV-16 E5, E6 and E7 genes [120] (b) Mice transgenic for 16E5 (without HPV 16 E6 and E7) develop skin tumours with high frequency [67].

One or more of the demonstrated biological activities of 16E5 may be responsible for early post-infection events enhancing the probability of subsequent carcinogenesis. Indeed viral persistence is considered to be an important factor in the neoplastic progression of a premalignant lesion [65]. The 16E5-induced down regulation of MCH I [104] and MHC II [107], resulting in the infected cell evading immunosurveillance, may increase the duration and size of the HPV infection raising the probability that some infected cells will become transformed. The expansion of infected cells may also be favoured by 16E5-induced DNA synthesis in differentiating keratinocytes [121] and by 16E5-enhanced ligand-dependent activation of EGF-R [56,58-60]. Furthermore the ability of 16E5 to inhibit gap-junction-mediated intercellular communication via connexin 43 interaction may render the infected cells more insensitive to homeostatic growth control signals from adjacent, non-infected cells [83,84]. 16E5 induction of cell fusion may represent another critical event in the early stage of HPV-associated cervical cancer [41-43]. Thus, HR HPVs E5 is involved in the very early stage of carcinogenesis by prolonging the life and expanding the pool of the infected cells from which cancer may arise as a stochastic event. Finally E5 can be also involved in negative selection of cells harboring episomal virus and in turn favoring the selection of cells with integrated virus. Episome loss, associated with induction of antiviral response genes, could be a key event in the spontaneous selection of cervical keratinocytes containing integrated HPV16. Microarray analysis showed that episome loss was closely associated with endogenous activation of antiviral response genes that are also inducible by type I IFN pathway [122]. Recently this activation of the antiviral state was shown to be induced by 16E5 through the stimulation of IRF-1 and IFN-β [123]. In this scenario cervical carcinogenesis requires not only HR HPV integration, but also loss of inhibitory (E2 expression) episomes by the E5-induced establishment of an antiviral state that may accelerate episomal clearance.

## BPV E5

BPV E5 is the best characterized of the E5 proteins and has provided the blueprint for investigations into HPV E5. E5 is the major BPV oncoprotein, only 44 amino acid in BPV type 1 (BPV-1) and 42 in BPV type 4 (BPV-4) [124-127]. Both proteins can be divided into two distinct domain: an amino-terminal domain which makes up the majority of the protein and consists of strongly hydrophobic leucine-rich membrane-spanning amino acid residues with a single hydrophilic amino acid at position 17 (glutamine in BPV-1 E5 and asparagine in BPV-4 E5), and a short hydrophilic carboxyl-terminal domain[125,128]. Both E5 proteins localize in the endomembrane compartments of the GA, ER and plasma membrane. It has been suggested for BPV-1 E5 that helix-helix hydrophobic contacts within the TM domain play a critical role in functional dimer assembly and that the cystein-containing motif functions as additional dimer stabilization [129-131].

### Cell transformation by BPV E5

Despite their structural similarity, BPV-1 and BPV-4 E5 differ in the ways they achieve cell transformation, likely reflecting the different origin of the cells hosting the two viruses. BPV-1 infects fibroblasts and skin keratinocytes giving rise to fibropapillomas, whereas BPV-4 infects solely the epithelial cells of the mucous epithelium of the upper gastrointestinal tract [132]. Thus, while expression of BPV-1 E5 by itself is sufficient to fully transform mouse and primary human fibroblast cells [133,134], BPV-4 E5 contributes to the transformed phenotype of primary cells by conferring anchorage independent growth, growth in low serum, focus formation and increased cell motility but only in the presence of the other viral oncoproteins, resembling in this respect 16E5 (Table 1) [127,135,136]. For both proteins the hydrophobic domain, the hydrophilic residues at position 17 and the hydrophilic C-terminal tail are critical for their transforming activities [135,137].

BPV-1 E5 induces tumourigenic cell transformation by strongly and specifically binding to its cellular target, Platelet Derived Growth Factor Receptor β receptor (PDGFβ-R) tyrosine kinase in a ligand-independent manner [138-141]. The PDGFβ-R is a transmembrane protein which is normally activated by binding of its ligand PDGF. BPV-1 E5 can bind as a dimer to two monomers of the PDGFβ-R [140,142,143], inducing receptor dimerisation which in turn causes trans-phosphyorylation of specific tyrosine kinase residues within the cytoplasmic domain of the receptor resulting in mitogenic signalling [144]. Studies using PDGFβ-R kinase inhibitors show that maintenance of transformation by BPV-1 E5 requires sustained PDGFβ-R activation [145,146].

BPV-1 E5 interacts with the PDGFβ-R via its transmembrane domain [141,142,147], unlike the natural ligand PDGF which binds the receptor via its ligand binding domain Extensive characterisation of E5 demonstrates that there are essentially four residues important for PDGFβ-R binding and activation. These are the transmembrane glutamine (Gln17) important for both dimerisation of E5 and for its interaction with PDGFβ-R; the aspartic acid (Asp 33) residue at the juxtamembrane region which provides a negative charge required for receptor interaction and two terminal cysteine residues (Cys37, Cys39) essential for homodimerisation of E5 [125,137,145,148,149]. Interestingly, these four residues are conserved among the E5 proteins of fibropapillomaviruses [150] whereas other residues within the transmembrane region are less well conserved. Mutagenesis studies have demonstrated that BPV-1 E5 can tolerate a surprising number of mutations and that the maintenance of the hydrophobic nature of E5 and the conserved residues is enough to confer transforming activity [151-155].

Mutational analyses of the receptor itself have identified key residues important for function. The PDGFβ-R is as single span type I transmembrane receptor with three domains: an amino terminal domain which binds its receptor, a short membrane spanning region and an intracellular kinase domain. Two residues appear to be important for E5 interaction and transformation activity: a threonine 513 in the transmembrane domain is required for H bonding with Gln17 residue of the E5 protein and lysine at 499 in the juxtamembrane region with Asp33 in the E5 protein [156-158]. An important feature of BPV-1 E5 mediated PDGFβ-R activation is that activation occurs independently of PDGF since E5 can constitutively activate PDGFβ-R deletion mutants that lack the extracellular ligand-binding domain [141,159].

The interaction between BPV-1 E5 and PDGFβ-R is highly specific. At physiological levels, E5 is able to interact with PDGFβ-R but not with other tyrosine kinase receptors including insulin receptors, basic fibroblast growth factor receptor or insulin-like growth factor receptors, however at higher levels E5 can interact with additional receptors [140,144]. E5 cannot bind the PDGF α receptor, a closely related receptor to the PDGFβ-R whilst both of these receptors can be activated by PDGF [140-144]. The specificity is thought to be conferred by the transmembrane domain [159].

**Figure 3:**
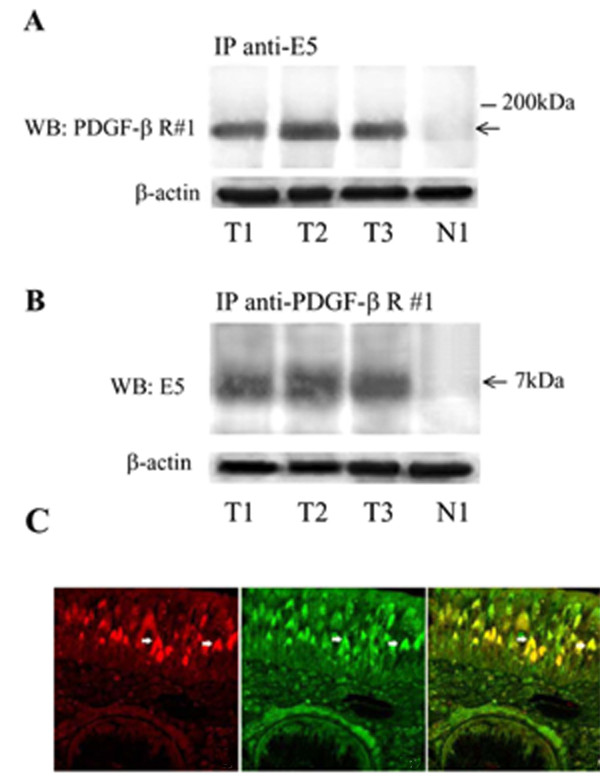
**BPV E5 and PDGFβ-receptor co-precipitate and co-localize in bovine urinary bladder carcinoma**. A. Co-immunoprecipitation of the PDGFβ-receptor with E5 antiserum. Tissue lysates were immunoprecipitated with the anti E5 antibody and the immunoblot was analyzed with the anti- PDGFβ-receptor antibody. Lanes 1-3 are BPV-2 positive bovine urinary bladder cancer samples, T1, T2, T3. Lane 4 is BPV-2 DNA positive normal bovine bladder mucosa, N1. B. Coimmunoprecipitation of the E5 oncoprotein with the PDGFβ-receptor antiserum. The arrow indicates the E5 oncoprotein and its estimated kDa weight. In a parallel blot, the same amount of lysate was probed with an antibody to β-actin to control for the quantity of protein (bottom panel) C. Colocalization (yellow) of PDGFβ-receptor (red) and E5 (green) in bovine neoplastic urothelium. The white arrows indicate a juxtanuclear position of E5 and PDGFβ-receptor (reproduced with permission from Borzacchiello et al., Oncogene 2006).

**Figure 4:**
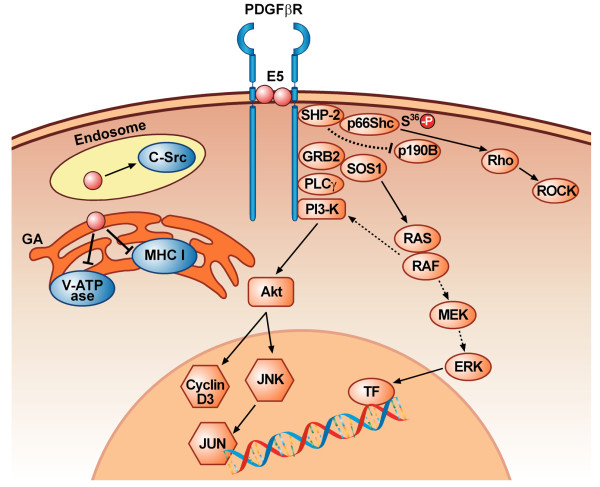
**Model of cellular events during cell transformation mediated by BPV-1 E5**. E5 binds to the transmembrane domain of PDGFβ-R and induces receptor dimerisation and autophosphorylation. The activated receptor can recruit p85-PI3K that activates the AKT and JNK pathway. PDGFβR stimulation of a PI3K-AKTpathway leads to increased expression of cyclin D3. Recruitment of Sos1-GRB2 complex to p-PDGFβR may activate Ras, although the downstream MAPK pathway is not activated; therefore Ras may activate PI3-K pathway. The SHP2-p66Shc complex bound to the activated PDGFβ-R may be recruited and inactivates p190BRhoGAP, resulting in activation of a Rho family GTPase, Rho A and its downstream effector ROCK inducing focus formation. E5 is able to interact with c-src in late endosomal and trans-Golgi compartment, thus resulting in c-src constitutively activation. E5 interacts with the C-terminus of MHC I heavy chain and causes the retention of MHC I in the Golgi apparatus, thus preventing its transport to the cell surface. E5 perturbs the physiological activity of the V-ATPase by inhibiting the correct assembly of V-ATPase and H+ pumping, thus inducing persistent alkalinization of the Golgi and endosomes vescicles.

Most of the studies on BPV E5 and PDGFβ-R activation have been performed in vitro. Borzacchiello et al (2006) have shown that BPV-2 E5 interacts with and activates the PDGFβ-R *in vivo *in bovine urinary bladder cancers (Figure 3). Moreover, the binding of E5 to PDGFβ-R induces the activation of different signal transduction pathways: PDGFβ-R and phosphoinositide 3 kinase (PI3K) physically interact as do PDGFβ-R and the Grb2-Sos complex. PI3K-Akt and Grb2-Sos-Ras signals are all potentiated in cancer, but, as in *in vitro *models, the levels of Erk and Mek proteins are not significantly overexpressed [160-162] (Figure 4). BPV E5 is able to activate c-src, a non receptor tyr kinase, but not the closely related c-Fyn and PI3K [163]. The activation of the latter is necessary but not sufficient to induce cell transformation independently of PDGFβ-R signalling [163,164]. Unlike BPV-1 E5, BPV-4 E5 induces transformation of established cells independently from the constitutive activation of tyrosine kinase growth receptors (Table 1) [165]. Cell transformation by BPV-4 E5 (either of established cells by itself or of primary cells in co-operation with other viral oncoproteins) is achieved through transactivation of the cyclin A gene promoter, increased cyclin A expression and cyclin A-associated kinase activity, and inhibition of the negative regulator of cell cycle, p27^Kip1 ^[135,166-168]. Mutational analyses of BPV-4 E5 have demonstrate that, as is the case for BPV-1 E5, the residue at position 17 and the hydrophilic C-terminal tail are critical for its transforming activities [135]. Thus both mutation of Asp 17 and deletion of the C-terminus hydrophilic domain completely abrogate cell transformation and E5 ability to activate the cyclin A pathway [135].

### Down-regulation of MHC expression

The down-regulation of surface MHC I and retention of the MHC I complex in the GA were first shown in cells expressing BPV-4 E5 [169] and then confirmed for other E5 proteins both from BPV and HPV. BPV E5 interferes with MHC I biosynthesis at multiple steps: transcriptional inhibition of the MHC class I heavy chain gene, degradation of the heavy chain and physical interaction with any residual heavy chain [169,170]. Only the physical interaction with heavy chain is shared with 16E5 [99]. Moreover, in the case of BPV-4 E5 the down-regulation of surface MHC I is irreversible, as interferon cannot restore the transport of the MHC I complex to the cell surface [128], while this is not the case for 16E5. This may reflect the fact that E5 is a "stronger" protein in BPV than in HPV.

In any event, the finding of MHC down-regulation by E5 has proved of great importance since it defined a new biological activity shared by both BPV and HPV E5 (Table 1), which allows the infected cell not to be effectively recognised by cytotoxic CTL [106].

### BPV E5: other biological activities

BPV E5 interacts with the 16 kDa protein, a component of the V-ATPase [147,171]. In the case of BPV-1 E5, this protein association exists as a tri-component complex, which contains also PDGFβ-R molecules. Mutational analyses have demonstrated that glutamic acid 143 in the transmembrane domain of the 16 kDa protein and glutamine 17 in the transmembrane domain of BPV-1 E5 are essential for E5-16 kDa stable association [172]. The binding of the E5 with 16 kDa perturbs the correct assembly of V-ATPase and H+ pumping, with persistent alkalinization of the GA and endosomal vesicles [103] and may lead, directly or indirectly, to a marked loss of cell-cell communication through down-regulation of gap junctions (Table 1) [101,173], rendering the transformed cells more insensitive to homeostatic growth control signals from adjacent normal cells. However, in the case of HPV-16 inhibition of gap junction intercellular communication has been attributed to the interaction of E5 with connexin 43 [84]. E5-expressing bovine urinary bladder tumours show activated calpain 3, suggesting a possible involvement of this protein in urothelial carcinogenesis [174].

### Targeting HPV-16 E5 for cervical treatment

16E5 activates many different cellular pathways involved mostly in the early stage of cervical carcinogenesis [175]. Different studies have pointed out the possibility of E5 targeting for CaCx therapy. In animal studies, 16E5 delivered by an adenovirus vector reduces the growth of tumours and the E5 vaccine induces protection against tumours through CD8^+ ^cytotoxic T cells (CTLs) [176]. The same research group has further demonstrated that the stimulating epitope was an E5 peptide (aminoacids 25-33) and vaccination with this peptide carrying CpG oligonucleotides reduced tumour growth [177]. 16E5 modulates different cellular pathways and the targeting of these relevant pathways may lead in a near future to a possible therapeutic approach. In addition, E5 is more consistently expressed in the early stage of viral infection and in precancerous lesions [178], and therefore E5 or E5 altered pathways could be targeted to cure the infection and to prevent precancerous lesions from progressing into invasive cancers. 16E5 interacts with many different pathways including upregulation of growth factor signalling, induction of inflammatory cell signalling, angiogenesis and antiapoptosis (Figure 2). Furthermore E5 participates in malignant transformation by supplementing (added value) the roles of E6 and E7. All these E5-induced signalling pathways (Figure 2) or E5 itself can be addressed by therapies already utilised against the other HPV oncogenes such as radioimmunotherapy, oncolytic adenoviruses [179], gene silencing using short interfering RNA (siRNA) [180] and others, which have been originally used to target the E5-upregulated pathways. EGF-R, COX-2 or ET1 inhibitors should be useful in controlling E5 activity in the precancerous lesions as well as other small molecules interfering with downstream molecules. Finally, E5 may be considered a viroporin, like the VP4 of SV40 [181] and, therefore, susceptible of therapy by compounds that affect similar structures produced by other viruses. Indeed amantidine, amiloride, long-alkyl-chain iminosugar derivatives and new compounds seem to affect, to various extent, the activity of the 16E5 protein in vitro (A. Macdonald, personal communication, 11 DNA TV Meeting, Trieste, Italy, 2011)

## Conclusions

The main activities of E5 can be resumed as follows:

Although E6 and E7 provide the primary transforming activities of HR HPVs, E5 can augment their function and contribute to tumour progression:

When expressed alone, HPV E5 has weak transforming activity.

In transgenic mouse models, 16E5 expression in the skin produces epithelial hyperproliferation with spontaneous tumour formation, whereas in estrogen-treated mice, expression of E5 alone can induce cancers [182], suggesting a role for E5 as a true oncoprotein.

The presence in HPV 16-positive cervical tumours of viral episomes in addition to viral integrants leads to the hypothesis that there are multiple pathways to HPV induced tumourigenesis see above. Beside the basic hypothesis of high-level expression of E6 and E7 consequent to the abrogation of the repressive effects of E2 due to the loss of the E2 gene during integration, another pathway can be active in cells that still maintain viral episomes, in which E5 would augment the activity of E6 and E7. The expression of E5 is increased on differentiation to promote proliferation of differentiated cells and productive viral replication.

The localization of HPV E5 to the endoplasmic reticulum suggests its activity may be related to the trafficking of cytoplasmic membrane proteins through this cellular compartment, in particular of growth factor receptors and of molecules involved in immune control.

There are multiple documented intracellular binding targets for 16E5 such as the EGF receptor family member ErbB4 [77], the 16-kDa subunit of the vacuolar H^+^-ATPase [39,55], the heavy chain of HLA type I [104], calnexin [51], the zinc transporter ZnT-1, the EVER1 and EVER2 transmembrane channel-like proteins [115,117] the nuclear import receptor family member KNβ3 [45], BAP31 and A4 [44,52] (Figure 2).

However the role of 16E5 in carcinogenesis seems to be limited to the early stages of cervical carcinogenesis because the E5 gene is frequently deleted when the HPV genome is integrated during malignant progression [28,68,183]. Nevertheless, the expression of 16E5 as detected by immunohistochemistry, was reported in approximately 80, 90 and 60% of HPV 16-infected LSILs, high-grade SILs and cervical carcinomas, respectively [28]. Furthermore data from a limited number of patients were presented at the 1^st ^International Workshop on E5 Oncogene [184] suggesting that E5 expression may lead to a worse response to treatment with taxanes (Venuti, unpublished data). Therefore, targeting E5 which is frequently expressed in earlier stages of malignant transformation may be a rational approach for preventing premalignant lesions from progressing into invasive cervical cancers [175]. The recent successful eradication of HPV-induced tumours in mice by anti-E5 vaccination [176] indicates that E5 can be used in immunotherapy. The neutralization of E5 or of E5-induced signalling pathways, therefore, whether immunological or chemical, could be advantageous particularly in HPV infections and pre-cancerous lesions where there are no treatments available.

## List of abbreviations used

HR: High Risk; PV: Papillomavirus; 16E5: HPV-16 E5; CxCa: cancer of the uterine cervix; PAP test: Papanicolau test; E: early proteins; L: late structural proteins; ORF: open reading frame; LSIL: low grade squamous intraepithelial lesions; CIN: cervical intraepithelial neoplasia; GA: Golgi apparatus; ER: endoplasmic reticulum; KNβ3: karyopherin β3; MHC I: Major histocompatibility complex class I; MHC II: Major histocompatibility complex class II; HLA: human leukocyte antigen; CTL: cytotoxic T Lymphocyte; EGF-R: epidermal growth factor receptor; NK: natural killer; MAPK: mitogen-activated protein kinase; PKC: protein kinase C; ET_A_: endothelin receptor; ET-1: endothelin-1; CKI: cyclin-dependent protein kinase inhibitors; TRAIL: Tumor necrosis factor-related apoptosis-inducing ligand; DISC: Death-Inducing Signalling Complex; FasL: Tumor necrosis factor ligand superfamily member; COX-2: cyclooxygenase 2; PGE_2_: Prostaglandin E2 receptor; XBP-1: X-box-binding protein 1; IRE: Serine/threonine-protein kinase/endoribonuclease IRE1; IFN: interferon; CREB: Cyclic AMP-responsive element-binding protein; PDGFβ-R: Platelet Derived Growth Factor Receptor β subunit receptor; PI3K: phosphoinositide3 kinase; V-ATPase: vacuolar H+-ATPase.

## Competing interests

The authors declare that they have no competing interests.

## Authors' contributions

All authors have contributed equally to the paper. All authors read and approved the final manuscript.
